# Brain functional changes in tibetan with obstructive sleep apnea hypopnea syndrome: A resting state fMRI study

**DOI:** 10.1097/MD.0000000000018957

**Published:** 2020-02-14

**Authors:** Dongjie Kang, Zongyuan Qin, Wen Wang, Yun Zheng, Huiying Hu, Yuanyuan Bao, Haihua Bao

**Affiliations:** Department of Medical Imaging Center, Affiliated Hospital of Qinghai University, Xining, China.

**Keywords:** amplitude of low-frequency fluctuation, functional connection, obstructive sleep apnea hypopnea syndrome, regional homogeneity, Tibetan, voxel-based morphology

## Abstract

Tibetan is a major ethnic group living on the Qinghai-Tibet Plateau in China. Due to their high-altitude hypoxia environment, sleeping disorder and obstructive sleep apnea hypopnea syndrome (OSAHS) are more prone to occur. In this study, we investigated the brain structural and functional differences between Tibetans OSAHS patients and Tibetans healthy controls using high resolution three-dimensional T1 weighted magnetic resonance imaging (MRI) and resting state functional MRI. The analysis was based on voxel-based morphology, regional homogeneity (ReHo), amplitude of low-frequence fluctuation (ALFF) and functional connection (FC) methods. A total of 14 OSAHS patients and 16 healthy control, all Tibetan male, matched closely in terms of age, education and living altitude, were recruited. The relationship between the ReHo and ALFF values at different brain areas and clinical features, including the apnea hypopnea index (AHI) in the OSAHS group, was analyzed using Pearson correlation. Compared with healthy control, OSAHS patients showed no significant gray matter volume or FC change. OSAHS group showed significantly increased ReHo values in the superior frontal gyrus dorsolateral, the left middle frontal gyrus, and the superior frontal gyrus medial. In contrast, OSAHS group showed decreased ReHo value in the left fusiform gyrus and cerebellum lobule 6. OSAHS group showed significantly increased ALFF values in the right inferior frontal gyrus orbital part, the right median cingulate and paracingulate gyri, the right Inferior frontal gyrus triangular part, the right insula and the left superior frontal gyrus dorsolateral. In the OSAHS group, the AHI showed a positive correlation with the ReHo value at the left cerebellum lobule 6 (r = 0.562, *P* = .037). Tibetan OSAHS patients had no significant change in brain structure and FC, which may be due to their adaption to the hypoxia environment. ReHo values and ALFF values changes in multiple brain areas in Tibetan OSAHS patients indicated brain functional impairment in multiple brain regions. The left cerebellum lobule 6 gradually compensates brain function as OSAHS progresses.

## 1 Introduction

Obstructive sleep apnea hypopnea syndrome (OSAHS) is the most common respiratory sleep disorder in the clinic. Its most noticeable feature is intermittent hypoxia caused by apnea. Tibetans living in the Qinghai-Tibet Plateau are prone to have OSAHS due to high altitude and hypoxia environment, where the residents may easily suffer the respiratory rate and pulse rate increases, low arterial oxygen saturation, poor sleep, and significant sleep-disordered breathing pattern.^[1]^ High-altitude environment can also lead to fatigue due to chronic hypoxia, memory loss, executive function reduction and work inefficiency, which can be indications of damages to brain function. A number of studies have concluded that OSAHS patients have structural and functional changes in multiple brain regions, and cognitive functions such as patient execution and memory have been reduced by varying degrees.^[2,3]^

Lorenzo et al^[4]^ pointed out that the Tibetan ethnics has different egg-laying deficiency protein nine-like protein 1 gene encoding prolyl hydroxylase domain-containing protein 2 from the Han ethnics, which contributes to the adaptive adjustment of hypoxia. In addition, Peng et al^[5]^ found that the down-regulation of endothelial pas domain-containing protein 1 is a molecular basis for Tibetans to adapt to low oxygen at high altitude. Ge et al^[6]^ found that long-term exposure to hypoxic conditions lead to an increase in oxygen transport through a comparative study of Tibetan and Han adults. In addition, different lifestyles can be expected to result in brain morphology changes.^[7]^ Due to their strong adaptability to the hypoxic environment and special lifestyles, Tibetans may have different brain structure and functional impairments resulted from OSAHS than other ethnic groups.

To fully understand the brain structural and functional changes in Tibetan OSAHS patients, we applied voxel-based morphology (VBM) on high resolution brain structural images and resting state fMRI for brain functional. VBM is a technique that can quantitatively analyze the entire brain structure with high objectivity and accuracy.^[8]^ Resting state fMRI is a imaging method with post-processing analysis for brain function analysis, its basic principle is to use the blood oxygen level dependent contrast to detect changes in neuronal activity, which can be assessed by regional homogeneity (ReHo) and amplitude of low-frequency fluctuation (ALFF). In certain brain regions, ReHo is positively correlated with the temporal consistency of neuronal activity, and ALFF is positively correlated with the spontaneous activity of neurons.^[9]^ Posterior cingulate cortex (PCC) is considered to be an important node in the default network of the resting state, so PCC is often selected for the analysis of the functional connection (FC) of the seed point.^[10]^

So far, studies of brain structure and function changes in Tibetan OSAHS patients have not been reported yet, so it is of great significance to study the neuropathophysiological mechanism of Tibetan brain alteration in the hypoxic environment. In this work, we studied the relationships between the ReHo and ALFF values at different brain regions and clinical features in the Tibetan OSAHS patients, and whether the Tibetan OSAHS patients showed alterations in gray matter volume (GMV), white matter volume (WMV), ReHo, ALFF and FC compared to the healthy Tibetan.

## 2 Participants and methods

### 2.1 Participants

All study subjects are Tibetan Chinese. 14 OSAHS patients diagnosed by polysomnography (PSG) and 16 healthy volunteer were recruited in this study. The diagnostic criteria of OSAHS were as follows: PSG monitored apnea hypopnea index (AHI) ≥5 times/h, accompanied by daytime sleepiness and other symptoms. The exclusion criteria were as follow:

(1) Abnormal blood pressure, blood lipids and blood glucose;
(2) Previous brain parenchymal injury or positive nervous system examination;
(3) History of mental or neurological disorders;
(4) History of treatment with drugs and surgery for OSAHS;
(5) Serious cardiovascular and respiratory diseases;
(6) Thyroid disease or other related diseases.

The research protocol was approved by the Medical Ethics Committee of the Affiliated Hospital of Qinghai University. All participants provided an informed consent form.

### 2.2 Respiration testing

All participants were monitored by PSG (Philips, Alice 6.), which recorded a polysomnogram of at least 7 hours from 21:00 pm to 7:00 am the next day. Simultaneous monitoring was performed for EEG, electro-oculogram, mandibular electromyography, electrocardiogram, fingertip pulse oximeter to monitor pulse oxygen saturation, mouth and nose pressure and heat-sensitive airflow, chest, and abdomen movement, body movement, leg movements, snoring, and other indicators. All sleep monitoring results were analyzed and interpreted by 2 experienced professional sleep technicians according to update of the 2007 American Academy of Sleep Medicine diagnostic criteria.^[11]^

### 2.3 Image acquisition

Structural and functional images of each subject were acquired on a Philips 3.0T TX Achieva magnetic resonance imaging (MRI) systems (Philips Medical, Amsterdam, Nederland). T1W-3dimensional structural MRI was acquired for each subject using a turbo field echo sequence (Repetition time/Echo time = 7.5/3.7ms, thickness = 2 mm, acquisition matrix = 256 × 256, flip angle = 7°), yielding 176 contiguous axial slices covering the whole brain and lasted 318 seconds. The resting state fMRI scan was acquired with an echo planar imaging sequence (Repetition time/Echo time = 2500/30ms, slice thickness = 3.5 mm, Field of View = 224 × 224 mm^2^, flip angle = 9°, 35 contiguous axial slices with 0.35 mm gap, scan time: 385 seconds) covering the whole brain. Earplugs and eye masks were provided for the comfort of each subject. During resting state fMRI, each subject was instructed to not have any thoughts during the examination, eyes closed and lying still on the scanner table.

### 2.4 Magnetic resonance imaging data processing

The three-dimensional T1 weighted images were processed using VBM8 software (http://www.neuro.uni-jena.de/vbm/) based on MATLAB 2010b (MathWorks, Natick, Massachusetts). Preprocessing consisted of the following steps:

(1) convert the digital imaging and communications in medicine format of the original image into the Neuroimaging Informatics Technology Initiative format.
(2) All MRI images were aligned with the anterior commissure (AC) and posterior commissure (PC) lines on the transverse plane, after motion correction, the structural images were segmented into white matter (WM), gray matter (GM) and cerebrospinal fluid.
(3) The individual WM and GM components were normalized into the standard Montreal neurological institute (MNI) template with a voxel size of 1.5 × 1.5 × 1.5 mm^3^ and modulated for GM and WM volumes using the diffeomorphic anatomical registration through exponential lie algebra approach.
(4) Smoothing with a Gaussian filter of 8 mm Full width at half maximum.

The resting-fMRI data were analyzed using the Statistical Parametric Mapping software (SPM8; http://www.fil.ion.ucl.ac.uk/spm/) implemented in MATLAB. Preprocessing included the following steps:

(1) Data preprocessing is performed using DPARSF software (http://rfmri.org/DPARSF/) to remove the first 10 time points of each resting state fMRI scan. Data exclusion: subject head motion exceeded 1.5 mm in any direction or head rotation exceeded 1.5 degree. Data for the remaining 140 time points were normalized to the MNI standard brain template. Re-sampling use the voxel of 3 × 3 × 3 mm^3^, smoothing with a Gaussian filter of 6 mm Full width at half maximum.
(2) ALFF analysis: The pre-processed data are de-linearly drifted and filtered by the ALFF tool from REST software, and the power spectrum of the signal of 0.01 to 0.08 Hz is squared to obtain the value of ALFF, which is normalized by mean ALFF from whole brain to obtain standard ALFF.
(3) ReHo analysis: The pre-processed data are calculated using the ReHo tool from the REST software to obtain Kendall coefficient concordance (KCC). The standardized ReHo is equal to the KCC value of each voxel divided by mean KCC from all voxels of the whole brain.
(4) The PCC is involved in memory formation, sensory monitoring and stereotypes. At the same time, previous resting-state fMRI studies have confirmed that the PCC is an important node of the default network.^[10]^ The PCC is chosen as the seed node to calculate the change of the FC of the default mode network (DMN). PCC selected DMN-ROIS template provided by Stanford University FIND lab website.^[12]^ The correlation between the PCC and the whole brain voxel time series is analyzed for the data after linear drift and filtering, and the correlation coefficient *r* is calculated and converted to *Z* value that matches the Fisher *Z* normal distribution.

### 2.5 Statistical analysis

All data are indicated as mean ± standard deviation. Brain function connectivity data processing in the whole brain mask was used as a template and the body mass index (BMI) was used as a covariate. One-sample *t* test was performed on the 2 groups (*P* < .01, cluster size >74 voxels AlphaSim correction). Then, 2 sets of independent sample *t* tests (*P* < .05, cluster size >389 voxels AlphaSim correction) were performed with BMI as a covariate, and 2 sets of data single-sample *t* test union results were selected as the mask of 2 independent sample *t* tests. Two sample *t* test were performed to compare the GMV, WMV, ReHo and ALFF values between the 2 groups using SPM8 (*P* < .05, cluster size >389 voxels AlphaSim correction). Furthermore, Pearson correlation was used to evaluate the relationship between ReHo and ALFF in differences brain regions in the OSAHS group with AHI index using SPSS, (SPSS Inc.,version 19.0,Chicago,Illinois). *P* value less than .05 were considered statistically significant.

## 3 Results

General information of the participants and clinical measurements are listed in Table 1.

**Table 1:**
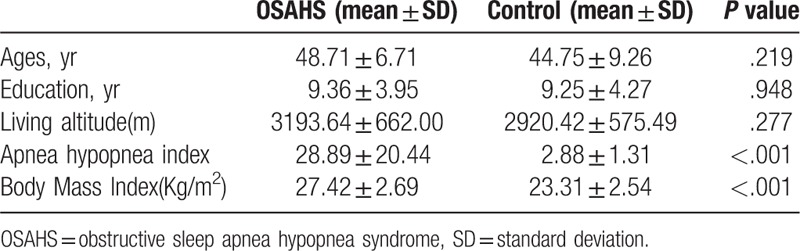
General information and clinical measurements of the 2 groups.

### 3.1 VBM

Between the OSAHS group and the healthy control group, there is no significant difference for the GMV and WMV value (*P* > .05).

### 3.2 ReHo

**Figure 1:**
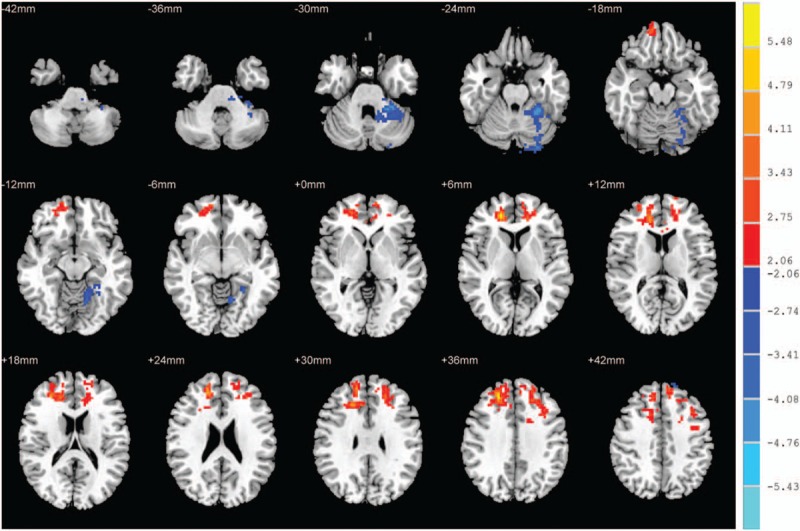
ReHo alterations in OSAHS group when compared to control group. Significantly increased ReHo values in the superior frontal gyrus dorsolateral, the left middle frontal gyrus, the superior frontal gyrus medial. Significantly decreased ReHo values in the left fusiform gyrus and cerebellum lobule 6. (cluster > 389 voxels, *P* < .05, AlphaSim corrected). Red indicates ReHo increase; Blue indicates ReHo decrease. OSAHS = obstructive sleep apnea hypopnea syndrome, ReHo = regional homogeneity.

Compared with control subjects, OSAHS patients showed significantly increased ReHo values in the superior frontal gyrus dorsolateral, the left middle frontal gyrus, and the superior frontal gyrus medial, and significantly decreased ReHo values in the left fusiform gyrus and left cerebellum lobule 6 (Table 2). The regions with significant ReHo changes in OSAHS patients are shown in Fig. 1.

**Table 2:**
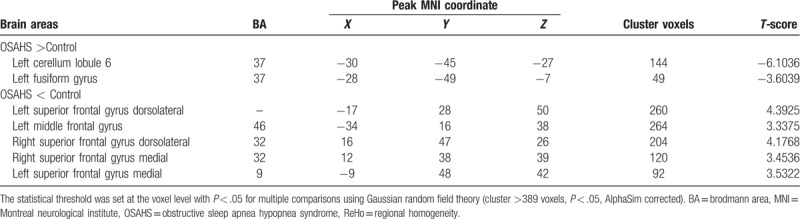
Brain areas with significantly different ReHo between the 2 groups.

### 3.3 ALFF

**Figure 2:**
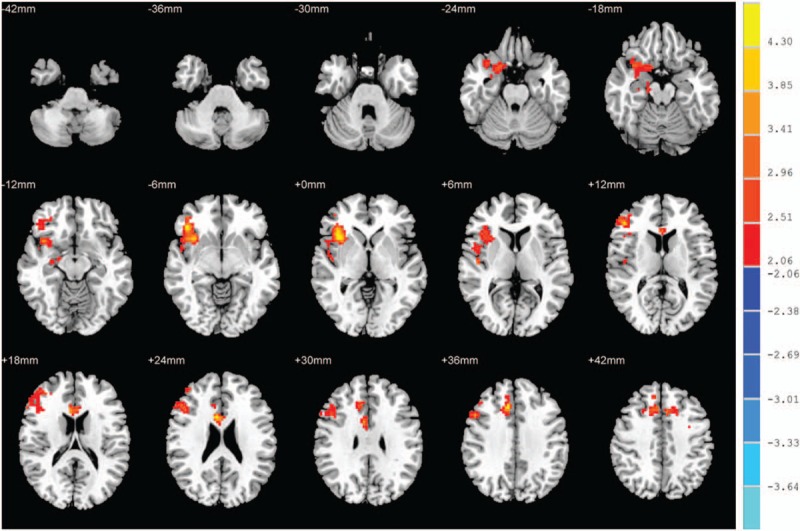
ALFF alterations in OSAHS group. Significantly increased ALFF values in the right inferior frontal gyrus orbital part, the right median cingulate and paracingulate gyri, the right inferior frontal gyrus triangular part, the right insula, and the left superior frontal gyrus dorsolateral (cluster >389 voxels, *P* < .05, AlphaSim corrected); ALFF = amplitude of low-frequency fluctuation, OSAHS = obstructive sleep apnea hypopnea syndrome.

Compared with control subjects, OSAHS patients showed significantly increased ALFF values in the right inferior frontal gyrus orbital part, the right median cingulate and paracingulate gyri, the right Inferior frontal gyrus triangular part, the right insula, and the left superior frontal gyrus dorsolateral (Fig. 2 and Table 3). We did not find any significantly decreased ALFF values in the OSAHS group (*P* > .05).

**Table 3:**
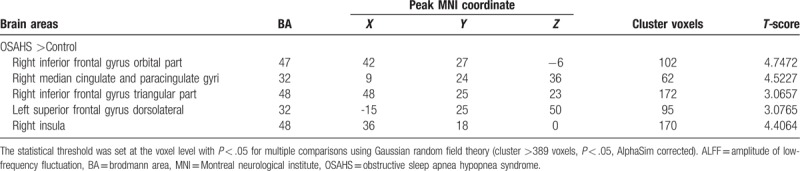
Brain areas with significantly different ALFF values between the 2 groups.

### 3.4 Functional connectivity

Compared to control group, the OSAHS group did not have any brain area with enhanced or weakened FC, that was associated with the PCC (*P* > .05).

### 3.5 Correlation analysis

**Figure 3:**
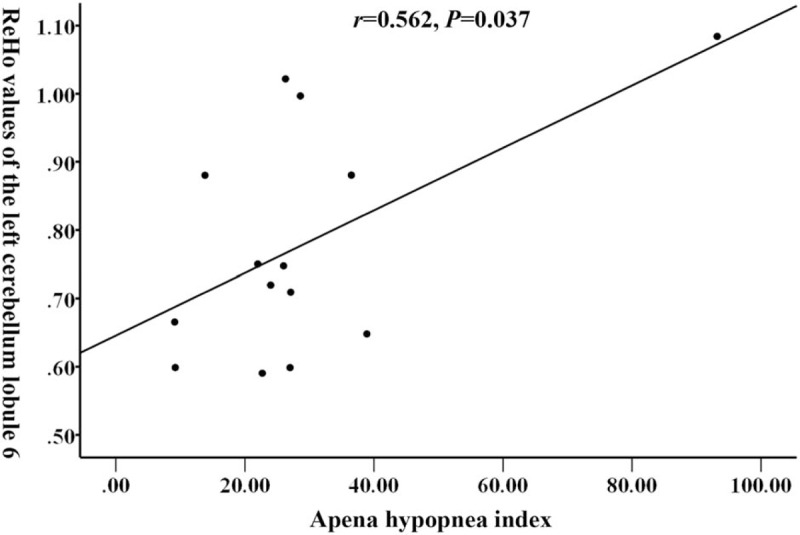
The AHI index of OSAHS patients was positively correlated with the mean ReHo values at the left cerebellum lobule 6 (*r* = 0.562, *P* = .037). AHI = apnea-hypopnea index, OSAHS = obstructive sleep apnea hypopnea syndrome, ReHo = regional homogeneity.

In the OSAHS group, the AHI index was positively correlated with the ReHo value at the left cerebellum lobule 6 (*r* = 0.562, *P* = .037; Fig. 3).

## 4 Discussion

### 4.1 Analysis of no significant differences region the GMV and function connection of Tibetan OSAHS group

This study used VBM technology to compare the brain structure of the OSAHS patients with those of the healthy volunteers in the Tibetan population. We found no significant changes in gray matter, white matter or CSF, which is consistent with the results from O’Donoghue et al.^[13]^ Yan^[14]^ used the VBM method and found that the GMV in the brain regions such as the bilateral forehead islands of the plateau people was lower than that of the plain people. These confirmed the pathophysiological mechanism of adaptation of the plateau aborigines to chronic hypoxia and compensation is different from that of people in the plains. In addition, the language and lifestyle difference between Tibetans and other nationalities may lead to certain brain structure differences.^[15]^

The OSAHS group did not have any brain regions that had abnormal functional connectivity to PCC. Chen^[16]^ found that although the DMN of the OSAHS patients met the small world network attributes, but OSAHS patients had abnormal FC in multiple brain regions and the topological properties of such abnormal default networks were related to cognition. Another study found that the FC between the right hippocampus and the posterior cingulate gyrus of the OSAHS patients was significantly weakened and considered to be the basis of cognitive dysfunction in OSAHS patients.^[17]^ Although the natural environment of the plateau and OSAHS has common hypoxia characteristics, Tibetans are more resistant and adaptive to hypoxic. We speculate that Tibetan adult brains have strong adaptability to hypoxia and compensatory capacity, the hypoxia increase in OSAHS patients did not result in further damage to the structural and FC of the brain.

### 4.2 Analysis of the ReHo value alteration in the Tibetan OSAHS group

This study found that the Tibetan patients with OSAHS had higher ReHo values in the left frontal gyrus, bilateral dorsolateral superior gyrus and medial frontal gyrus than the ReHo values in the same regions from the healthy Tibetan subjects. The ReHo values in the left fusiform gyrus and cerebellum 6 region decreased in OSAHS patients. The Tibetan OSAHS patients AHI index was significantly positively correlated with ReHo value at the left cerebellum 6 regions. Santarnecchi^[18]^ found that the ReHo values of OSAHS patients in 14 brain regions such as bilateral anterior wedge anterior lobe were reduced, while the ReHo values of 9 brain regions including the right sacral gyrus and cortex were increased, which is similar to our finding. Peng^[19]^ also found changes in ReHo values in multiple brain regions in OSAHS patients. The most significant ReHo change in our study is located in the frontal lobe. The increase in ReHo value indicates that the function of this area is compensatory and positively activated. It has been found that the frontal lobe is most sensitive in a hypoxic environment and is significantly associated with working memory function.^[20]^ Long-term high altitudes may lead to decreasing cerebral oxygen saturation and functional changes in the frontal cortex. In this study, the ReHo value of the bilateral dorsolateral recurrent gyrus increased and its loss of function may result in the occurrence of mental illness, such as depression.^[21,22]^ In addition, some studies have found that the ReHo value of patients with depression in the left cerebellum 6 areas is reduced. The cerebellum has certain function to force the patient to re-inhale after the apnea occurs. When this function is impaired, the patient will have breathing difficulty again and even wake up.^[23,24]^

### 4.3 Analysis of the ALFF value alteration in the Tibetan OSAHS group

We found that the Tibetan OSAHS group had increased ALFF value in the inferior temporal gyrus of the right side of the island, the medial and lateral sacral gyrus, the triangular inferior gyrus, the insula, and the left lateral dorsal gyrus. These areas with increased ALFF indicate that the spontaneous activity of the nerves in these brain regions is enhanced. Li^[25]^ found that the ALFF values of patients with OSAHS in the right anterior wedge and bilateral posterior cingulate gyrus were reduced, while the ALFF value of the left inferior frontal gyrus was increased. Behavioral outcome-based resting state functional MRI (rs-fMRI) studies have shown that associative memory function correlates with ALFF values in the frontal region.^[26]^ Zhang^[27]^ showed that the ALFF value of the insular lobe decreased in the hypoxic state, which may be related to the reduction of ventilation driving force. While our study found that the ALFF value of the right insula increased, which indicates the Tibetans’ strong adaptive capability, that is, they can improve the ventilation function to adapt to the state of hypoxia.

Our study has several limitations. First, the overall sample size is relatively small. Second, lack of psychological and cognitive function tests. Further investigation is needed with increased sample size and a complete cognitive test.

## 5 Conclusion

In this study, VBM, ALFF, ReHo, and FC were utilized to investigate the brain structure and function changes of Tibetan OSAHS patients in the high altitude area. Brain functional and structural differences between Tibetan OSAHS patients and Tibetan normal volunteers were studied. Using rs-fMRI, we found the cerebellum 6 regions is negatively activated, while the other brain regions are positively activated. This result from the Tibetan OSAHS patients is not completely consistent with previous studies of OSAHS patients, which may be due to the physiological characteristics of Tibetans living in the high-altitude hypoxic environment.

In addition, our results indicate that Tibetan OSAHS patients have functional damage in multiple brain regions and the function of the left cerebellar 6 region is gradually compensated as the OSAHS condition worsens. The Tibetan OSAHS patients did not have significant brain structure and FC change, which may be related to the adaption to the long-term hypoxic environment before they suffered OSAHS.

## Author contributions

**Conceptualization:** Dongjie Kang, Haihua Bao.

**Data curation:** Dongjie Kang.

**Formal analysis:** Dongjie Kang.

**Funding acquisition:** Huiying Hu, Haihua Bao.

**Investigation:** Dongjie Kang, Zongyuan Qin.

**Methodology:** Zongyuan Qin, Huiying Hu, Haihua Bao.

**Project administration:** Zongyuan Qin.

**Software:** Yun Zheng.

**Supervision:** Wen Wang, Yuanyuan Bao, Haihua Bao.

**Validation:** Wen Wang.

**Visualization:** Dongjie Kang, Zongyuan Qin, Yuanyuan Bao.

**Writing – original draft:** Dongjie Kang.

**Writing – review and editing:** Haihua Bao.
